# *Ceratonia siliqua*: Phytochemistry, Bioactivity, and Its Emerging Role as a Sustainable Food and Industrial Resource

**DOI:** 10.1111/1750-3841.71527

**Published:** 2026-09-30

**Authors:** Anis Ben Hsouna, Ravendra Kumar, Manjul Gondwal, Shivangi Chamoli, Bhanu Pratap Singh Gautam, Simranpreet Kaur, Rakesh Kumar Verma, Piyush Kumar, Monika Michalak, Boutheina Ben Akacha, Wirginia Kukula‐Koch, Wojciech Koch, Faical Brini, Wissem Mnif, Rania Ben Saad, Stefania Garzoli, Miroslava Kačániová

**Affiliations:** ^1^ Laboratory of Biotechnology and Plant Improvement Centre of Biotechnology of Sfax Sfax Tunisia; ^2^ Department of Environmental Sciences and Nutrition, Higher Institute of Applied Sciences and Technology of Mahdia University of Monastir Monastir Tunisia; ^3^ Department of Chemistry, College of Basic Sciences and Humanities G. B. Pant University of Agriculture and Technology Pantnagar India; ^4^ Department of Chemistry Radhey Hari Govt. P. G. College, Kashipur Kashipur India; ^5^ Department of Biotechnology Graphic Era Deemed to be University Dehradun India; ^6^ School of Health Sciences and Technology UPES, Bidholi Campus Dehradun India; ^7^ Department of Zoology Laxman Singh Mahar Campus (Affiliated to Soban Singh Jeena University, Almora) Pithoragarh India; ^8^ School of Health Sciences and Technology UPES, Bidholi Campus Dehardun India; ^9^ Department of Dermatology, Cosmetology and Aesthetic Surgery, Collegium Medicum Jan Kochanowski University Kielce Poland; ^10^ Department of Pharmacognosy with Medicinal Plants Garden Medical University of Lublin Lublin Poland; ^11^ Department of Food and Nutrition Medical University of Lublin Lublin Poland; ^12^ Department of Chemistry, College of Science University of Bisha Bisha Saudi Arabia; ^13^ Department of Chemistry and Technologies of Drug Sapienza University Rome Italy; ^14^ Institute of Horticulture, Faculty of Horticulture Slovak University of Agriculture Nitra Slovakia; ^15^ School of Medical & Health Sciences VIZJA University Warszawa Poland

**Keywords:** bioactive compounds, carob tree, *Ceratonia siliqua*, functional food, health benefits, industrial applications, phytochemistry

## Abstract

*Ceratonia siliqua* L. (*C. siliqua*), commonly known as the carob tree, is an evergreen species of the Fabaceae family native to the Mediterranean basin and increasingly cultivated worldwide. Traditionally valued as animal feed and a natural sweetener, it has recently gained recognition as a multifunctional plant resource with nutritional, pharmaceutical, and industrial relevance. This review synthesizes current knowledge on the botany, phytochemistry, nutritional profile, bioactivity, and traditional uses of *C. siliqua*, and discusses its economic and industrial significance. Emphasis is placed on its rich content of polyphenols, flavonoids, dietary fiber, proteins, and essential minerals, which have been associated with antioxidant, antidiabetic, antidiarrheal, antimicrobial, and cardiometabolic‐related activities. Beyond its nutritional potential, carob is increasingly used in food processing, functional foods, and pharmaceutical formulations, with locust bean gum as a key industrial derivative. Despite its long history, recent advances highlight new applications and the need for systematic quality control, valorization of genetic resources, and sustainable exploitation strategies. Overall, this review provides an integrative perspective that bridges traditional knowledge with modern scientific findings, aiming to guide future research and support broader utilization of carob as a natural bioresource in health, nutrition, and industry.

## 1 Introduction

*Ceratonia siliqua* L., commonly known as the carob tree, is a perennial evergreen species of the Fabaceae family, traditionally cultivated in the Mediterranean basin and increasingly introduced into various agro‐ecological zones worldwide (Dahmani et al. 2025). For centuries, it has served as a source of food and animal feed and has held symbolic and economic significance in many Mediterranean societies. Its importance is now being re‐evaluated in the context of climate change, food security, and the need for sustainable resources that support health. Carob's resilience to arid climates, low water requirements, and ability to thrive in poor soils underscore its potential as a climate‐resilient crop and a strategic species for sustainable agriculture (Viruel et al. 2020).

In recent years, *C. siliqua* has attracted renewed attention due to its alignment with sustainability goals and the global transition towards plant‐based, drought‐resilient crops that support environmental and nutritional security (Brassesco et al. 2021; Martins‐Loução et al. 2024).

From nutritional and pharmaceutical perspectives, carob has been highlighted as a nutrient‐rich food source and a reservoir of high‐value bioactive compounds. Its pods, seeds, and leaves contain dietary fiber, polyphenols, flavonoids, and essential minerals, which have been linked to antioxidant, antimicrobial, antidiabetic, antidiarrheal, anti‐obesity, and cardiometabolic‐related activities (Aydın and Özdemir 2017). This interest aligns with the growing demand for plant‐based diets, natural preservatives, and functional foods. Industrially, carob has also gained traction: locust bean gum (LBG, E410), derived from the seed endosperm, is widely used as a thickening, stabilizing, and emulsifying agent in the food, pharmaceutical, and cosmetic sectors. These ecological, nutritional, and industrial dimensions illustrate why *C. siliqua* has become a subject of growing scientific and societal interest (Battle et al. 1997; Aydın and Özdemir 2017; Dahmani et al. 2025).

Despite significant progress, current research remains fragmented. Many studies have focused on specific aspects—such as phytochemical characterization, pharmacological activities, or industrial processing—without integrating these findings into a unified understanding of the species. Furthermore, variability in phytochemical composition among cultivars, the lack of standardized analytical and extraction methods, and limited clinical validation of pharmacological claims represent major gaps in the literature (Laaraj et al. 2023; Hssaini et al. 2024).

Therefore, this review aims to synthesize and critically analyze recent advances (2010–2025) in the botany, phytochemistry, nutritional composition, bioactivity, and traditional uses of *C. siliqua*, as well as its industrial applications and economic relevance. By integrating ecological, biological, and industrial perspectives, it seeks to consolidate the state of the art and identify current challenges and opportunities for innovation.

By providing a cross‐disciplinary synthesis, this review bridges traditional ethnobotanical knowledge with modern scientific findings, positioning *C. siliqua* as a versatile and sustainable bioresource with potential relevance to food security, public health, and the circular bioeconomy in the context of climate adaptation (Viruel et al. 2020).

## 2 Methodology

The present review was based on a comprehensive survey of the scientific literature available on *Ceratonia siliqua*. To ensure that the collected information was both relevant and reliable, the literature search was planned before data collection and conducted according to the main recommendations of the PRISMA (Preferred Reporting Items for Systematic Reviews and Meta‐Analyses) 2020 Statement (Page et al. 2021), adapted to the objectives of this narrative review.

The bibliographic search was performed using five international databases: Scopus, Web of Science, ScienceDirect, PubMed, and Google Scholar. Publications available until March 2026 were considered. Since several landmark studies published before 2010 remain essential for understanding the phytochemistry, nutritional composition, and biological properties of *C. siliqua*, no lower publication date limit was imposed. The search combined terms including *Ceratonia siliqua*, *carob*, *phytochemistry*, *bioactive compounds*, *nutritional composition*, *biological activities*, *functional food*, *traditional uses*, *industrial applications*, and *climate resilience*. Different combinations of these terms were generated using the Boolean operators AND and OR. In addition, the reference lists of the selected articles were examined to identify further relevant publications that had not been retrieved during the electronic search.

Only articles published in English and directly related to the objectives of this review were considered. Original research papers formed the core of the literature, while review articles were included to provide broader scientific context where appropriate. Conference papers containing sufficient methodological information were also considered. Editorials, duplicated records, abstracts without accessible full text, and publications not directly addressing *C. siliqua* were excluded.

The selection process involved several successive steps. After completion of the database search and manual screening of reference lists, 250 publications were retrieved. Duplicate records were removed before titles and abstracts were examined for relevance. The remaining articles were then evaluated through full‐text reading according to the predefined eligibility criteria. At the end of this process, 103 publications were retained and used for the preparation of this review. A summary of the study selection procedure is provided in the PRISMA flow diagram.


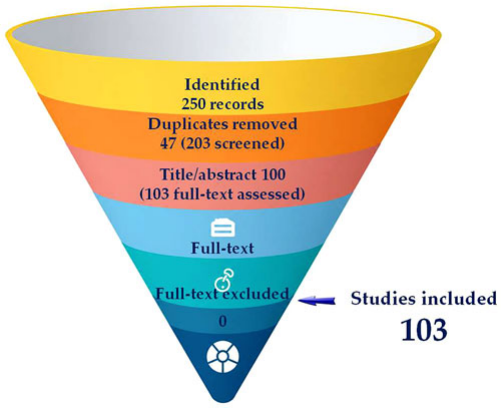


**PRISMA flow diagram**

For each selected publication, information relevant to the objectives of the review was extracted, including the publication year, geographical origin of the study, plant material investigated, and the principal findings. The collected literature was subsequently organized into thematic sections covering botanical aspects, phytochemical composition, nutritional characteristics, biological activities, traditional uses, and industrial applications of *C. siliqua*. Rather than simply compiling published results, the available evidence was critically examined to identify areas of agreement, inconsistencies among studies, and aspects requiring further investigation. Particular attention was also given to differences related to plant origin, cultivar, extraction procedures, and experimental approaches, as these factors may explain part of the variability reported in the literature.

## 3 Botanical Characterization and Distribution

The taxonomic classification of *C. siliqua* L. is summarized below to provide a concise botanical overview based on authoritative sources, including *Flora Europaea* (1968) and recent systematic updates (Martins‐Loução et al. 2024). This section clarifies the taxonomic position of the species within the Fabaceae family and highlights its ecological and morphological significance (Viruel et al. 2020). The valid scientific name is *C. siliqua* L., first described by Linnaeus in *Species Plantarum* (1753). The botanical classification of *C. siliqua* according to *Flora Europaea* (1968) is presented below:

**Table:** 

Taxonomic rank	Classification
Kingdom	Plantae
Subkingdom	Tracheobionta
Division	Magnoliophyta
Class	Magnoliopsida
Order	Fabales
Family	Fabaceae
Subfamily	Caesalpinioideae
Genus	Ceratonia
Species	*Ceratonia siliqua* L.

Under the pre‐molecular classification systems still reflected in some older floras, Ceratonia was historically treated within the segregate family Caesalpiniaceae; the Legume Phylogeny Working Group (LPWG) (2017) reclassification resolved this into a single, molecularly supported family Fabaceae with an expanded Caesalpinioideae, the framework adopted throughout this review (Ringelberg et al. 2022).

*C. siliqua* L., commonly known as the carob tree, is a perennial evergreen species belonging to the Fabaceae family, subfamily Caesalpinioideae. It is a slow‐growing, long‐lived tree that typically reaches heights of 6–10 m and is well adapted to semi‐arid Mediterranean climates characterized by mild winters and hot, dry summers (Dahmani et al. 2025). The species exhibits high morphological variability, particularly in pod and seed traits, depending on genotype, geographical origin, and environmental conditions (Viruel et al. 2020).

The natural distribution of *C. siliqua* encompasses the Mediterranean basin, ranging from Southern Europe (Spain, Italy, Greece) to North Africa (Morocco, Algeria), and extending into parts of the Eastern Mediterranean or West Asia (Turkey, Cyprus) (Plants of the World Online, 2026; Dahmani et al. 2025). From the Mediterranean basin, it was then introduced to several locations in Africa (Angola, Kenya, and Zimbabwe), Asia (China, Yemen, Iraq, Pakistan, and India), Europe (Germany and Portugal), North America (Trinidad and California), South America (Peru), and Australia (New South Wales) (Plants of the World Online, 2026). *C. siliqua* typically grows in well‐drained calcareous soils, tolerates salinity and drought, and plays an important ecological role in soil stabilization and reforestation programs (Mahdad and Gaouar 2023).

Figure 1 illustrates the global distribution of *C. siliqua* based on Food and Agriculture Organization of the United Nations (FAO) GeoNetwork datasets. The classification of distribution areas as native or introduced follows FAO GeoNetwork definitions, where native areas correspond to regions in which the species is considered indigenous according to historical biogeographical records, while introduced areas refer to regions where *C. siliqua* has been deliberately or accidentally introduced outside its natural range for cultivation or agroforestry purposes. The map was prepared using ArcGIS Pro 3.1 software and depicts recorded native and introduced populations between 2000 and 2023 (Hussain et al. 2011; Mahdad and Gaouar 2023).

**FIGURE 1:**
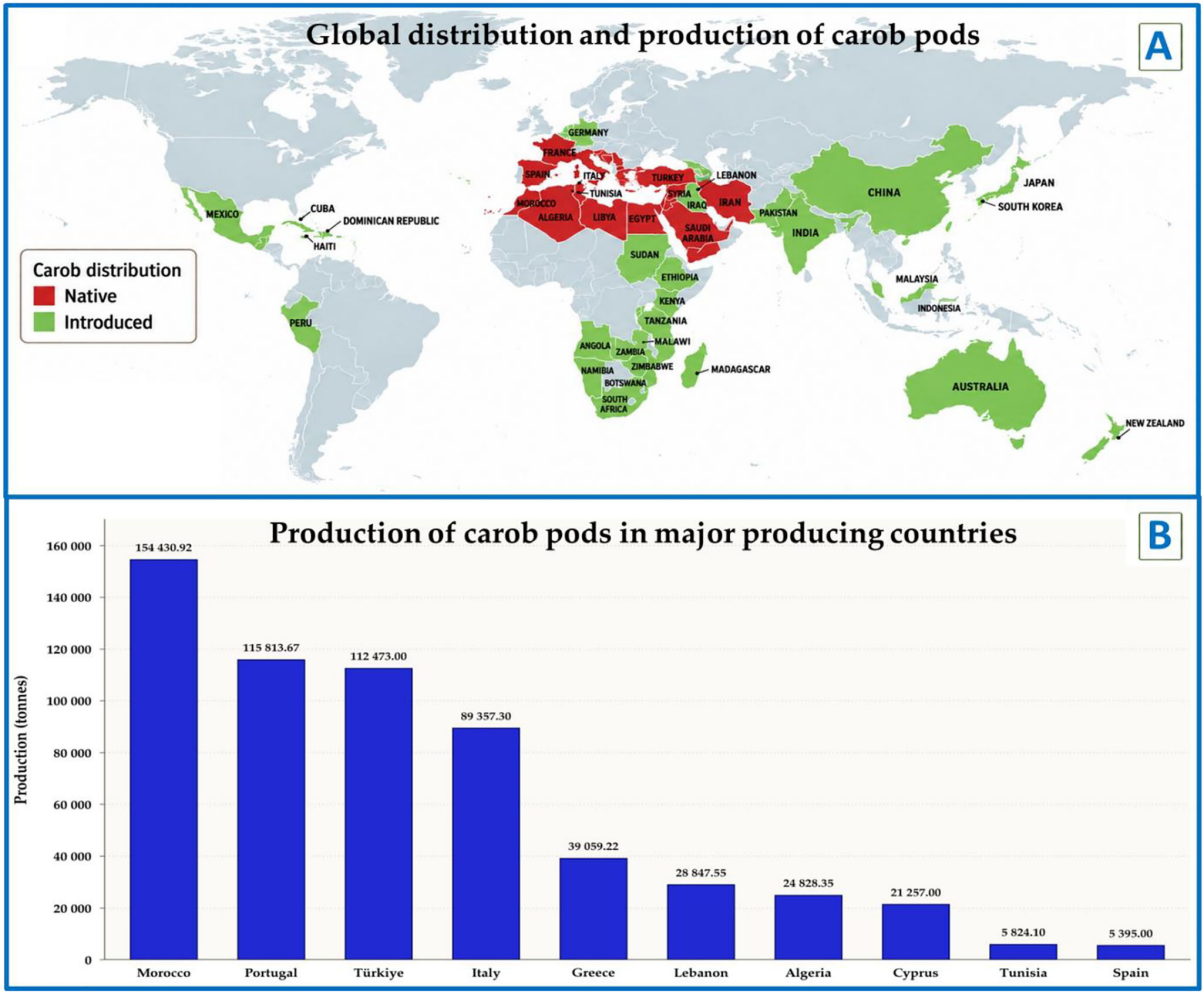
(A) Global distribution map of *C. siliqua* based on FAO GeoNetwork data. Native and introduced distribution areas were classified according to FAO GeoNetwork definitions. The map represents recorded occurrences documented between 2000 and 2023 and was generated using ArcGIS Pro 3.1 (ESRI [Environmental Systems Research Institute], 2024). (B) Production statistics of top 10 global producers of carob beans (in tons).

## 4 Nutritional and Phytochemical Composition of *C. siliqua*

Carob contains a broad spectrum of nutritional and phytochemical constituents distributed across pods, seeds, leaves, and bark, which together underpin its nutritional, pharmacological, and techno‐functional attributes (Figure 2). These constituents are considered here in two parts: (i) the nutritional composition, comprising carbohydrates, dietary fiber, proteins, lipids, minerals, and vitamins; and (ii) the bioactive/phytochemical composition, comprising polyphenols, flavonoids, tannins, D‐pinitol, and related secondary metabolites.

**FIGURE 2:**
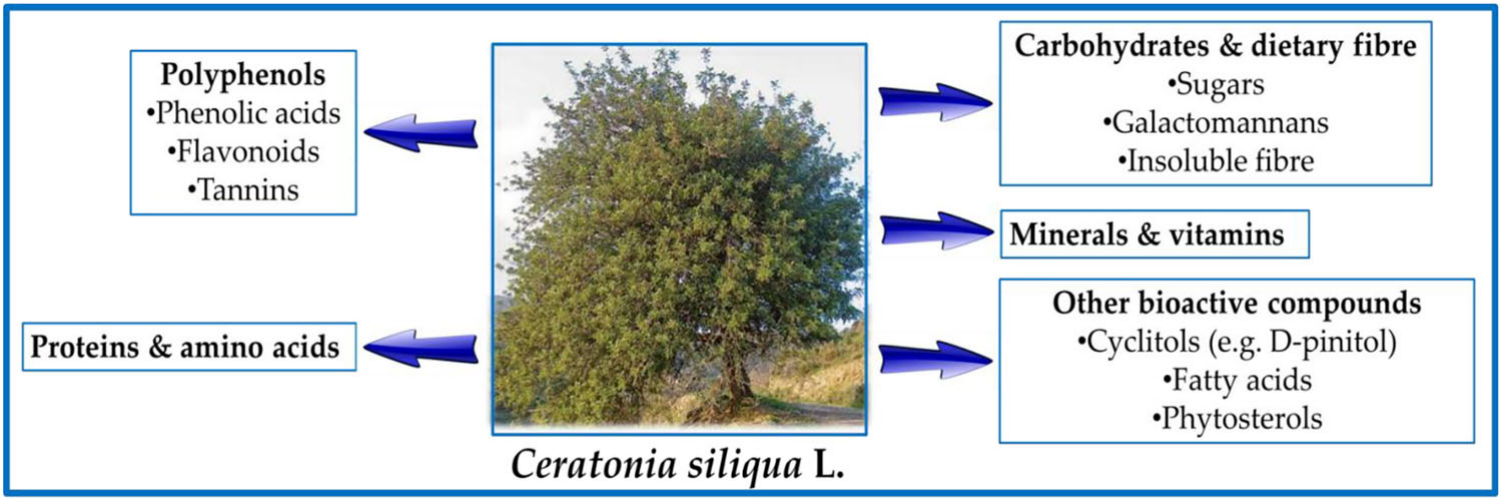
Schematic illustration of the major classes of bioactive compounds reported in *C. siliqua*. The figure provides a conceptual overview of the principal phytochemical groups identified across different plant organs based on literature data and is not intended to represent a quantitative, compositional, or comparative profile.

**TABLE 1:** Compositional and phytochemical overview of *C. siliqua* plant parts, encompassing both nutritional constituents (sugars, fiber, protein, and galactomannan) and phytochemical markers (polyphenols and tannins) (2024 update).

Plant part	Compound class	Key markers	Range / units (reported range)	Geographical origin	Analytical method	Reference
Pod pulp	Polyphenols / phenolic acids and flavonoids	Gallic acid, protocatechuic acid, catechin, epicatechin, quercetin derivatives	TPC typically 20–40 mg GAE/g DW in pod extracts	Egypt, Tunisia (Sfax)	HPLC‐DAD; LC–MS/MS; Folin–Ciocalteu	(Darwish et al. 2021; Dammak et al. 2024)
Leaves	Polyphenols / flavonoids	Kaempferol, tannic acid, catechin hydrate, polydatin	Flavonoids accounting for >70% of the total phenolic fraction in some extracts	Morocco	HPLC‐DAD; HPLC‐ESI‐MS/MS	(Meziani et al. 2015; Dahmani et al. 2025)
Seeds (whole)	Polyphenols / tannins	Catechin, epicatechin, condensed tannins	TPC ∼30–60 mg GAE/g DW depending on cultivar and extraction solvent	Turkey	HPLC‐DAD; spectrophotometric assays	(Fidan et al. 2020; Laaraj et al. 2023)
Pod pulp	Sugars and cyclitols	Sucrose, glucose, fructose, D‐pinitol	Total sugars 48%–56% DW; sucrose 65%–75% of total sugars; D‐pinitol up to ∼5%–7% DW or ∼50%–80 mg/g in syrup	Turkey (Antalya) Cyprus	HPLC‐RI; GC–MS; enzymatic sugar assays	(Turhan 2014; Christou et al. 2019; Nasrallah et al. 2024; Correia and Pestana 2024)
Pod pulp	Dietary fiber	Insoluble fiber (cellulose, hemicellulose, lignin); soluble fiber fractions	Total dietary fiber ≈ 18–25% DW; insoluble fraction predominates	NR	AOAC gravimetric fiber methods	(Brassesco et al. 2021; Darwish et al. 2021)
Seed endosperm	Galactomannans	Galactomannan (mannose: galactose ≈ 4:1)	Galactomannans 80–88% of endosperm DW	NR	Carbohydrate composition assays; HPAEC; colorimetric	(Brassesco et al. 2021; Nasrallah et al. 2024)
Seed embryo	Proteins and amino acids	Arginine, proline, leucine, other essential amino acids	Crude protein ≈ 50% DW of embryo; essential amino acids meeting FAO/WHO patterns	Italy(Sicily)	Kjeldahl N; amino acid profiling (HPLC)	(Avallone et al. 1997; Laaraj et al. 2023)
Pod pulp	Minerals	Potassium (K), calcium (Ca), magnesium (Mg), phosphorus (P), iron (Fe), manganese (Mn), zinc (Zn), and copper (Cu).	K typically 900–1100 mg/100 g DW; Ca ∼250–350 mg/100 g DW; other minerals in lower amounts	Turkey (Antalya)	ICP‐OES; AAS; ash mineral analysis	(Musa Özcan et al. 2007)
Seeds & pods	Cyclitol	D‐pinitol	Flesh: ∼53–55 mg/g DW; syrup: ∼65–78 mg/g; enriched extracts up to ∼9.7 g/L	Cyprus (Limassol)	GC–MS; HPLC; optimization of extraction	(Turhan 2014; Christou et al. 2019; Goulas and Georgiou 2020; Azab 2022)

Abbreviations: DW: dry weight; FAO/WHO: Food and Agriculture Organization/World Health Organization; GAE: gallic acid equivalents; GC–MS: gas chromatography–mass spectrometry; HPAEC: high‐performance anion‐exchange chromatography; HPLC‐DAD: high‐performance liquid chromatography with diode‐array detection; HPLC‐RI: high‐performance liquid chromatography with refractive‐index detection; LC–MS/MS: liquid chromatography–tandem mass spectrometry; NR: not reported. TPC: Total phenolic content.

### 4.1 Nutritional Composition

The carob fruit is a complex matrix of secondary metabolites. It contains a diverse range of nutrients, including dietary fiber, sugars, and various polyphenols (Table 1). In addition, carob is a source of several minerals and amino acids (Figure 3).

**FIGURE 3:**
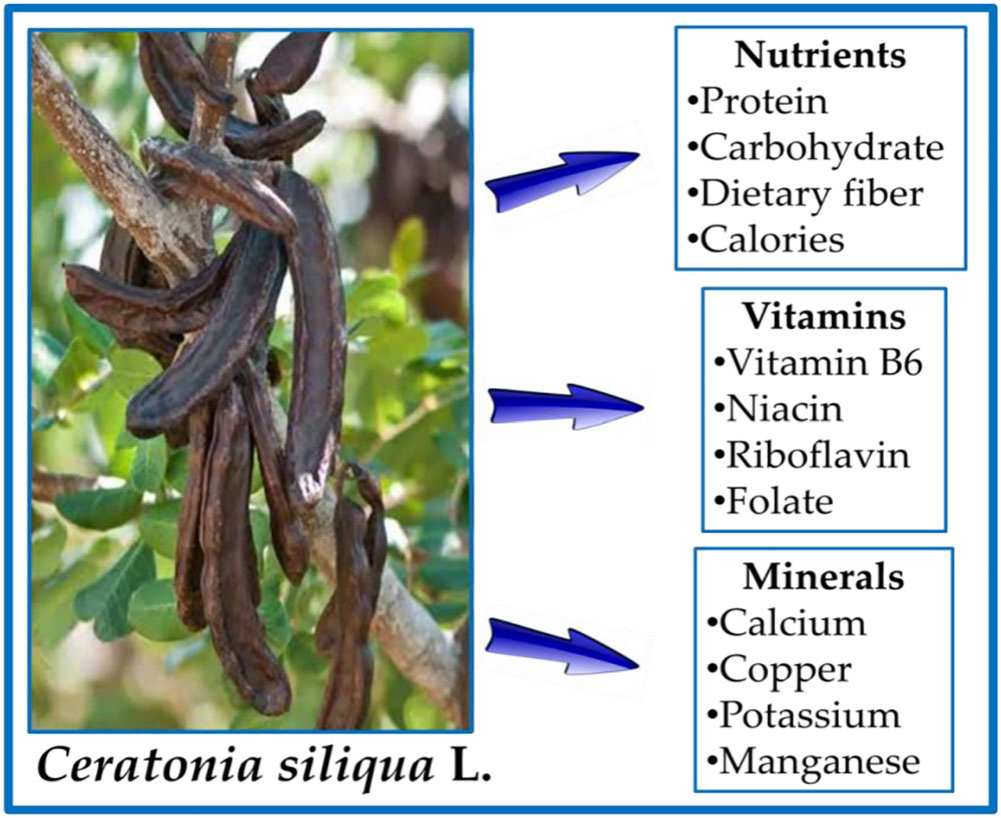
Illustrates the nutritional composition of carob and its associated health benefits.

The nutritional composition of *Ceratonia siliqua*, particularly its high content of dietary fiber, minerals, and bioactive compounds, is closely associated with its reported health benefits. These components contribute to various physiological effects, including glycemic regulation, digestive health, and cardiometabolic support. In this context, the discussion of macronutrients and culinary applications is relevant, as it reflects the practical pathways through which these health‐related effects may be achieved.

#### 4.1.1 Carbohydrates and Dietary Fiber

*C. siliqua* provides both soluble and insoluble dietary fiber, mainly lignin, cellulose, and hemicellulose, with smaller contributions from pectins and tannins, which have been associated with various physiological effects (Marco et al. 1997). Leaves, on a dry‐weight basis, contain carbohydrates, lipids, proteins, ash, and dietary fiber (Rtibi et al. 2017). Pods are characterized by high concentrations of sugars, including fructose, glucose, and sucrose, together with substantial fiber content (Palafox‐Carlos et al. 2011). Seeds are rich in sucrose and glucose and yield monosaccharides such as mannose, galactose, glucose, arabinose, and xylose following polysaccharide hydrolysis (Fidan et al. 2020).

Carbohydrates represent the predominant macronutrient fraction in *C. siliqua* pods, accounting for approximately 45–60% of dry weight (DW). The principal sugars include sucrose, glucose, and fructose, with sucrose being the most abundant, constituting up to 70–80% of total soluble sugars (Turhan 2014; Nasrallah et al. 2024). The carbohydrate profile is strongly influenced by cultivar, maturity stage, and growing conditions (Correia and Pestana 2024). Structural polysaccharides such as cellulose, hemicellulose, and lignin constitute the insoluble fiber fraction, while the endosperm is particularly rich in galactomannan, an economically important soluble fiber used in food and pharmaceutical applications (Brassesco et al. 2021).

#### 4.1.2 Proteins and Amino Acids

Carob provides relevant levels of proteins, including both essential and non‐essential amino acids. Seeds are particularly rich in arginine and alanine and contain moderate amounts of lysine, as well as isoleucine and valine (Fidan et al. 2020). In total, seventeen amino acids have been reported, including aspartic and glutamic acids, serine, glycine, histidine, threonine, alanine, tyrosine, valine, proline, methionine, isoleucine, leucine, cysteine, phenylalanine, and lysine (Ayaz et al. 2009).

Protein levels in *C. siliqua* vary considerably among plant tissues. The seed embryo exhibits the highest protein concentration, ranging from 45–55 g/100 g DW, whereas the pod pulp contains only 3–7 g/100 g DW (Avallone et al. 1997; Laaraj et al. 2023). The amino acid profile is characterized by relatively high levels of arginine, proline, leucine, and lysine, providing a balanced amino acid composition (Benyaich et al. 2025).

Proteins in the endosperm are mainly structural and associated with galactomannans, contributing to the seed's hardness and functional properties. Protein content and amino acid composition likewise vary with cultivar, environmental conditions, and fruit maturity (Benyaich et al. 2025).

#### 4.1.3 Lipids and Fatty Acids

Leaves from Tunisia exhibit lipid profiles dominated by unsaturated fatty acids, particularly linolenic (C18:3) and linoleic (C18:2) acids (Bonnet et al. 2019). Linolenic acid serves as a precursor of long‐chain polyunsaturated fatty acids such as EPA and DHA, which are relevant to neural and retinal development (Hsouna et al. 2011).

#### 4.1.4 Minerals and Vitamins

*C. siliqua* provides vitamins A and B, insoluble fiber, sugars, and a diverse range of minerals (Vekiari et al. 2011; Hsouna et al. 2011). In samples collected from Chefchaouen (northern Morocco), calcium was the most abundant mineral in leaves and bark, accompanied by substantial levels of potassium, magnesium, sodium, phosphorus, chlorine, copper, iron, zinc, and selenium. Bark was particularly rich in potassium, whereas leaves showed higher concentrations of calcium, magnesium, phosphorus, and zinc; bark contained higher levels of copper, iron, and selenium (El‐Hajaji et al. 2013).

Carob pods contain significant amounts of essential minerals, particularly potassium (K), calcium (Ca), and magnesium (Mg), along with trace elements such as iron (Fe), zinc (Zn), and manganese (Mn) (Musa Özcan et al. 2007; Fidan et al. 2020). Potassium concentrations typically range from 900–1100 mg/100 g DW, calcium from 250–350 mg/100 g DW, and magnesium from approximately 150–220 mg/100 g DW. Total dietary fiber content ranges between 18 and 25 g/100 g DW and is dominated by the insoluble fraction, while soluble fiber, mainly galactomannan, accounts for approximately 5–8 g/100 g DW (Brassesco et al. 2021; Darwish et al. 2021). The proximate composition of *C. siliqua* pods and seeds is summarized in Table 2.

**TABLE 2:** Nutritional composition of *C. siliqua*.

Component	Mean content (per 100 g DW)	Range	Analytical method	Geographical origin	Reference
Carbohydrates (total)	50.0 g	45–60 g	AOAC gravimetric method	NR	(Nasrallah et al. 2024)
Sucrose	30.0 g	25–35 g	HPLC‐RI	Turkey (Antalya)	(Turhan 2014; Correia and Pestana 2024)
Glucose	4.0 g	3–6 g	HPLC‐RI	Turkey (Antalya)	(Turhan 2014)
Fructose	3.0 g	2–5 g	HPLC‐RI	Turkey (Antalya)	(Turhan 2014)
D‐pinitol	4.5 g	2–6 g	GC–MS	Cyprus	(Christou et al. 2019; Goulas and Georgiou 2020)
Protein (pods)	5.0 g	3–7 g	Kjeldahl N	Italy (Sicily)	(Avallone et al. 1997)
Protein (seeds)	50.0 g	45–55 g	Kjeldahl N; HPLC amino acid profiling	NR	(Laaraj et al. 2023; Benyaich et al. 2025)
Total dietary fiber	22.0 g	18–25 g	AOAC enzymatic–gravimetric	NR	(Brassesco et al. 2021)
Soluble fiber (galactomannan)	6.0 g	5–8 g	AOAC 991.43	NR	(Brassesco et al. 2021)
Insoluble fiber	16.0 g	13–20 g	AOAC 991.43	Egypt	(Darwish et al. 2021)
Potassium (K)	1000 mg	900–1100 mg	ICP–OES	Turkey (Antalya)	(Musa Özcan et al. 2007)
Calcium (Ca)	300 mg	250–350 mg	AAS	Turkey (Antalya)	(Musa Özcan et al. 2007)
Magnesium (Mg)	180 mg	150–220 mg	ICP–OES	Turkey (Mersin)	(Fidan et al. 2020)
Iron (Fe)	5.0 mg	4–7 mg	ICP–OES	Turkey (Mersin)	(Fidan et al. 2020)
Zinc (Zn)	2.5 mg	2–3 mg	ICP–OES	Turkey (Antalya)	(Musa Özcan et al. 2007)

Abbreviations: AAS: atomic absorption spectrometry; AOAC 991.43: official AOAC method 991.43 for determining total, soluble, and insoluble dietary fibre; DW: dry weight; ICP–OES: inductively coupled plasma–optical emission spectrometry; NR: not reported; values represent mean or typical ranges reported in the cited studies from Mediterranean‐growing regions.

In Moroccan plant material, five macronutrients (potassium [K], calcium [Ca], chlorine [Cl], magnesium [Mg], and sodium [Na]) and thirty trace elements (e.g., iron [Fe], aluminium [Al], strontium [Sr], rubidium [Rb], copper [Cu], zinc [Zn], manganese [Mn], zirconium [Zr], barium [Ba], thorium [Th], bromine [Br], thulium [Tm], chromium [Cr], selenium [Se], cerium [Ce], lanthanum [La], caesium [Cs], antimony [Sb], neodymium [Nd], mercury [Hg], cobalt [Co], arsenic [As], scandium [Sc], molybdenum [Mo], tantalum [Ta], gold [Au], hafnium [Hf], samarium [Sm], silver [Ag], and europium [Eu]) were detected across different pod fractions (Elbouzidi et al. 2024).

### 4.2 Bioactive Compounds / Phytochemical Composition

#### 4.2.1 Polyphenols and Flavonoids

Polyphenols represent the major class of bioactive compounds in *C. siliqua* and are largely responsible for its antioxidant, anti‐inflammatory, and anticancer activities; they are primarily concentrated in pods, pulp, and leaves (Laaraj et al. 2023; Hssaini et al. 2024) (Figure 4). Leaves generally exhibit higher total phenolic, flavonoid, and condensed tannin contents than other plant organs (Custódio et al. 2011; El‐Hajaji et al. 2013). Across tissues, gallic acid is commonly detected in leaves, pulp, and seeds (Rtibi et al. 2017). Flavonols, including quercetin, myricetin, and kaempferol and their glycosides, predominate, with quercetin and myricetin rhamnosides frequently reported as the most abundant; flavones (apigenin, luteolin, and chrysoidin), flavanones (naringenin), and isoflavones (genistein) occur at lower levels (Owen et al. 2003; Papagiannopoulos et al. 2004; Ortega et al. 2009).

**FIGURE 4:**
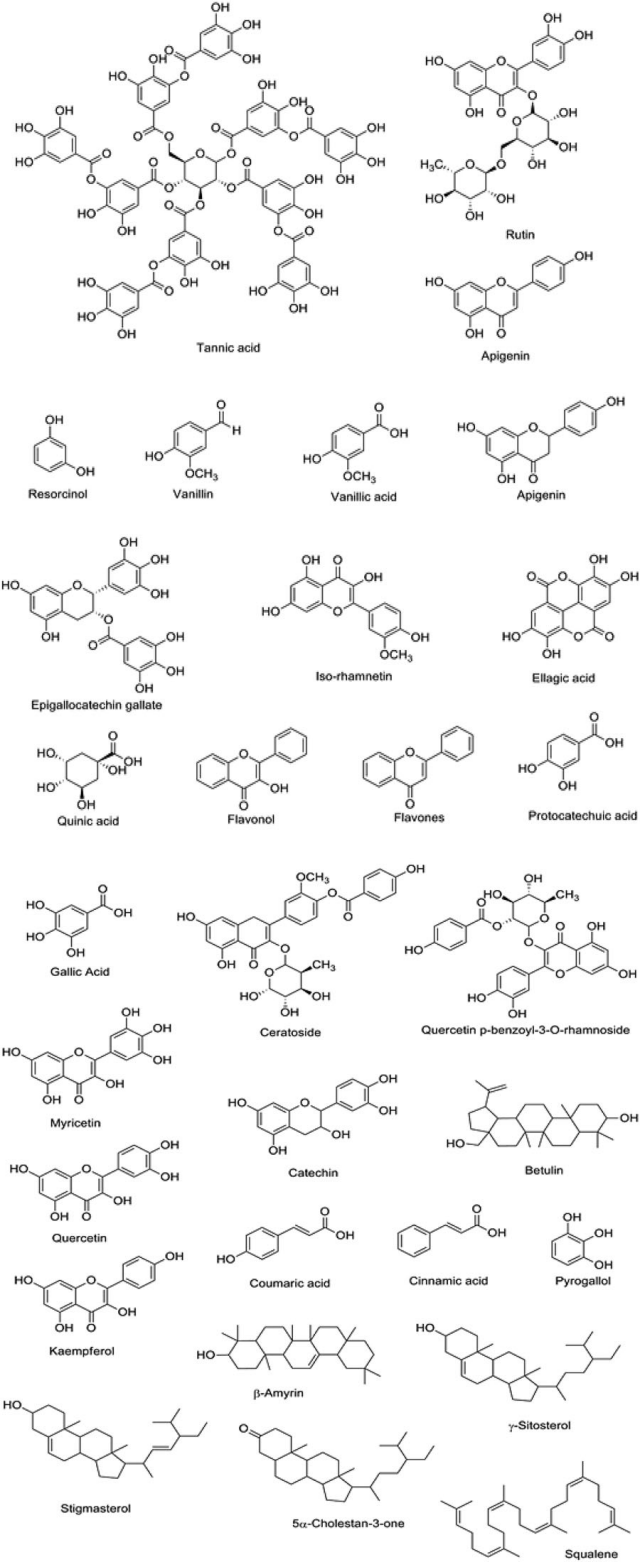
Selected chemical structures of bioactive compounds identified in *C. siliqua* according to published studies. These structures represent commonly reported constituents and do not reflect the full phytochemical diversity or relative abundance within the species.

Ultra‐high‐performance liquid chromatography (UPLC) and HPLC analyses have identified luteolin‐7‐glucoside, epicatechin, apigenin‐7‐glucoside, quercetin‐3‐*O*‐glucoside, caffeic acid, gallic acid, and chlorogenic acid in leaves (Abidar et al. 2020). In pods, polyphenolic profiles include quercetin glycosides, catechin, epicatechin, and epigallocatechin gallates, gallic and ellagic acids, and proanthocyanidins or ellagitannins; hydrolysable tannins such as gallotannins and ellagitannins have also been reported (Corsi et al. 2002; Ortega et al. 2009). Carob fiber contains multiple phenolic compounds dominated by gallic acid derivatives; simple phenols include cinnamic acid, while major flavonoids comprise myricetin glycosides and quercetin‐3‐*O*‐*L*‐rhamnoside (Owen et al. 2003). Depending on fruit maturity, pods may contain pyrogallol, catechin, tannic acid, gallic and chlorogenic acids, and epicatechin, whereas leaves also contain kaempferol, tannic acid, catechin hydrate, and polydatin (Rtibi et al. 2017). Overall, pods are particularly rich in polyphenols, flavonoids, and tannins, supporting reported health‐related effects, with notable constituents including gallic acid, catechol, pyrogallol, and chlorogenic acid (Yousef et al. 2024, Yousef et al. 2025).

In summary, the nutritional profile of *Ceratonia siliqua* supports its relevance as a bioactive plant matrix, with high levels of carbohydrates (notably sucrose and D‐pinitol) and dietary fiber (including galactomannans) being particularly relevant to its physiological and techno‐functional properties. Proteins and essential minerals (e.g., K, Ca, and Mg) further contribute to its nutritional value. Importantly, these primary constituents coexist with diverse secondary metabolites, including phenolic acids (gallic and chlorogenic acids), flavonoids (quercetin, myricetin, and kaempferol), and tannins (proanthocyanidins and gallotannins), suggesting that its biological effects are likely driven by synergistic interactions. However, this chemical complexity and variability remain a key limitation for standardization and mechanistic interpretation. Further studies integrating metabolomic profiling and standardized extraction approaches are required to better elucidate structure–activity relationships and to support its rational industrial and pharmacological exploitation.

#### 4.2.2 D‐Pinitol as a Bioactive Cyclitol

In addition to its role as a carbohydrate‐associated constituent quantified in Section 4.1.1, D‐pinitol is pharmacologically relevant in its own right, with reported insulin‐mimetic activity mediated through phosphoinositide 3‐kinase/protein kinase B (PI3K/Akt) signaling (see Section 5.3.2). Its dual nutritional–bioactive character exemplifies the conceptual overlap that can exist between primary and secondary metabolites in *C. siliqua*, and it is treated quantitatively under Section 4.1.1 to avoid duplication of compositional data.

In summary, the nutritional profile of *Ceratonia siliqua* supports its relevance as a bioactive plant matrix, with high levels of carbohydrates (notably sucrose and D‐pinitol) and dietary fiber (including galactomannans) being particularly relevant to its physiological and techno‐functional properties. Proteins and essential minerals (e.g., K, Ca, and Mg) further contribute to its nutritional value. Importantly, these primary constituents coexist with diverse secondary metabolites, including phenolic acids (gallic and chlorogenic acids), flavonoids (quercetin, myricetin, and kaempferol), and tannins (proanthocyanidins and gallotannins), suggesting that its biological effects are likely driven by synergistic interactions. However, this chemical complexity and variability remain a key limitation for standardization and mechanistic interpretation. Further studies integrating metabolomic profiling and standardized extraction approaches are required to better elucidate structure–activity relationships and to support its rational industrial and pharmacological exploitation.

### 4.3 Factors Affecting Compositional Variability

The nutritional and phytochemical composition of *C. siliqua* is not fixed but varies substantially according to at least five interacting factors, which collectively account for much of the heterogeneity.

#### 4.3.1 Cultivar and Genotype

Carob is propagated both from seed and through grafted cultivars, and considerable genotype‐dependent variability has been reported in sugar profile, D‐pinitol content, and polyphenol composition. D‐pinitol concentration alone has been shown to range from approximately 2 to 6 g/100 g DW depending on the cultivar (Christou et al. 2019; Goulas and Georgiou 2020), a more than threefold difference that has direct implications for antidiabetic potency. Despite this demonstrated importance, cultivar identity is inconsistently reported across the primary literature and is frequently absent from studies whose results are pooled in comparative tables; systematic documentation of cultivar alongside compositional data in future primary studies would substantially improve the comparability and interpretability of the field.

#### 4.3.2 Geographical Origin

Populations from different regions of the Mediterranean basin and beyond show marked differences in mineral and phenolic content, reflecting both genetic divergence and local soil chemistry. For instance, mineral profiles reported for Moroccan populations (El‐Hajaji et al. 2013) differ appreciably in trace‐element composition from values reported for other Mediterranean origins, underscoring that compositional data from one region cannot be assumed to generalize to carob grown elsewhere.

#### 4.3.3 Maturity Stage

Sugar and fiber composition change substantially as pods ripen sucrose and reducing‐sugar ratios shift over the course of maturation, and protein content in the seed embryo is influenced by the physiological maturity of the fruit at harvest (Correia and Pestana 2024; Benyaich et al. 2025). Harvesting at different stages of ripeness is therefore itself a source of the compositional variability reported across studies, independent of genotype or origin.

#### 4.3.4 Environmental Conditions

As a Mediterranean species adapted to semi‐arid climates, *C. siliqua* is exposed to considerable inter‐annual and inter‐site variation in temperature, water availability, and soil type, all of which influence secondary metabolite accumulation. Water‐deficit stress has been associated with altered sugar and phenolic profiles, consistent with the broader pattern whereby abiotic stress modulates polyphenol biosynthesis in Mediterranean tree crops (Viruel et al. 2020; Correia and Pestana 2024).

#### 4.3.5 Processing Methods

Post‐harvest handling, including drying temperature, duration, and roasting, further modifies the compositional profile, altering sugar caramelization, protein solubility, and the distribution of soluble versus insoluble fiber fractions (Nasrallah et al. 2024; Dammak et al. 2024). Roasting, commonly applied to improve flavor and shelf‐life, has been associated with reductions in native polyphenol content relative to unroasted pulp, illustrating a direct trade‐off between processing choices and the bioactive potential.

## 5 Health benefits of *C. siliqua*

### 5.1 Traditional Uses

Humans have utilized *C. siliqua* L. as a nutritional and therapeutic resource since antiquity, owing to its edible pods and versatile derivatives (Tardío et al. 2006; Bouaziz et al. 2013). Historical sources indicate that ancient Egyptians used carob pods as livestock feed and employed carob gum as an adhesive in mummy wrappings (Lentini and Venza 2007). In Arab cultures, the carob seed was adopted as a unit of weight, known as qirat or karat, which later became the standard for measuring gold and gemstones (Montesano et al. 2012).

Traditional uses of Ceratonia siliqua extend beyond medicinal applications to include a wide range of dietary practices across Mediterranean and Middle Eastern regions. Table 3 enumerates selected carob‐enriched dietary products, highlighting their incorporation into various traditional and modern food formulations.

**TABLE 3:** Carob‐ enriched functional foods and their impact on the quality of the product.

Country	Ethnomedicinal / food uses	Part and/or method of use	Reference
Morocco	Diarrhea/toxic for fish	Fruits, barks	(Coviello et al. 2015)
	Intestinal parasites, digestive system, antidiarrheal	Leaves, fruits, decoction or raw	(Liu et al. 2023)
	Diabetes	Seeds, leaves, or fruits infusion, powder, or decoction	(YAMAMOTO et al. 2000)
	Digestive diseases	Leaves, seeds, decoction or powder	(Son et al. 2010)
		Fruit powder	(Ennabili et al. 2000)
Tunisia	Food	Pulp	(El‐Hilaly et al. 2003)
	Gastrointestinal disorders, diarrhea	Fruits	(Ouhaddou et al. 2015)
Egypt	Diarrhea	Fruits, leaves infusion	(Nayak et al. 2015)
Jordan	Diabetes	Leaves decoction, cold or hot beverage of the pods	(Orch et al. 2020)
	Cough		(Baydoun et al. 2015)
Lebanon	Sweetener	Molasses of ripened fruits	(Ennabili et al. 2000)
	Food	Pods	(Ennabili et al. 2000)
	Medicine	Not indicated	(El‐Hilaly et al. 2003)
Palestine	Food	Raw fruits	(Ouhaddou et al. 2015)
	Hypertension	Raw or cooked fruits, seeds	(Ouhaddou et al. 2015)
Iraq	Abdominal pain, diarrhea	Fruits	(Afifi‐Yazar et al. 2011)
Turkey	Diuretic, purgative	Fresh fruits	(Hinad et al. 2022)
	Diarrhea	Leaves	(Vekiari et al. 2011)
Cyprus	Diabetes	Fruits decoction	(Baydoun et al. 2015)
	Laxative, demulcent	Pods	(Akça et al. 2020)
Greco‐Arab	Diabetes, herpes, lip sores	Leaves decoction	(Akça et al. 2020)
India	Hypocholesterolemic, hypolipidemic, hypoglycemic, demulcent, resolvent	Not indicated	(Fatima 2017)
Iran	Menorrhagia	Patient should sit in a pot of a decoction of several plants	(Tansaz et al. 2016)
Spain	Chocolate or coffee substitute, preservative for olives	Fruits, leaves	(Tardío et al. 2006)
Sicily	Food	Raw or boiled fruits	(Aissani et al. 2012)
Italy	Emollient	Decoction with *Ficus carica L*. and *Malva sylvestris L*.	(Montesano et al. 2012)

Throughout the Mediterranean Basin, the sweet pulp of carob fruits has long been consumed as food and animal feed, often mixed with barley flour. Carob flour has also been used in piglet feed due to its palatability and nutritional value. Over time, *C. siliqua* has gained recognition for its medicinal properties. Traditional medicine reports a wide range of uses, including anti‐inflammatory, antibacterial, antidiarrheal, antioxidant, antiulcer, antitussive, and hypoglycemic applications (Mekhoukhe et al. 2018). Roasted seeds and carob syrup have traditionally been used to relieve cough, sore throat, and wart lesions.

In Palestine, infusions prepared from carob pods and seeds are used to alleviate hypertension and digestive disturbances (Tardío et al. 2006). In Tunisia, decoctions and carob juice are applied to treat gastrointestinal disorders and diarrhea (Lentini and Venza 2007). In Morocco, leaves and seeds are administered in traditional formulations for diabetes management, whereas in Turkey, leaves are used as antidiarrheal remedies and the wood is valued for producing slow‐burning charcoal (Montesano et al. 2012; Mekhoukhe et al. 2018) (Table 4). These practices illustrate the deep cultural integration of carob within Mediterranean ethnopharmacology and its dual role as both food and medicine.

**TABLE 4:** Ethno‐medicinal and food uses of carob (*C. siliqua* L.).

Country / region	Plant part used	Traditional use	Mode of preparation / administration	Reference
Egypt	Pods, gum	Used in livestock feed; gum employed as adhesive in mummy wrappings	Direct feed / topical adhesive	(Lentini and Venza 2007)
Arab world	Seeds	Used as weight unit (*qirat or karat*) for gold and gemstones	Measurement standard	(Montesano et al. 2012)
Palestine	Pods, seeds	Hypertension, digestive disturbances	Decoction or infusion of crushed pods/seeds	(Tardío et al. 2006)
Tunisia	Pods, juice	Gastrointestinal disorders, diarrhea	Oral juice or aqueous decoction	(Lentini and Venza 2007)
Morocco	Leaves, seeds	Diabetes management	Infusion / decoction for oral use	(Montesano et al. 2012)
Turkey	Leaves, pods	Antidiarrheal; roasted seed powder for cough and throat irritation	Infusion, roasted powder mixed with honey	(Mekhoukhe et al. 2018)
Mediterranean region (general)	Pods, syrup	Cough, sore throat, wart treatment, tonic	Syrup or roasted flour	(Mekhoukhe et al. 2018; Nasrallah et al. 2024; Dahmani et al. 2025)

However, most of the available evidence is based on in vitro and animal studies, and further well‐designed clinical studies are required to confirm these effects in humans.

### 5.2 Culinary Applications of Carob

Currently, carob seeds are used as a substitute for cocoa powder in culinary products, and their chemical and sensory characteristics have been analyzed in depth (Hssaini et al. 2024). The substitution of cocoa in white milk chocolate with carob pod powder is under investigation. Protein content increased slightly with higher levels of carob powder substitution, while fiber and sugar contents increased significantly.

The most pronounced effect was a gradual reduction in caffeine content, which disappeared entirely at a 100% substitution level of carob powder. Aroma showed no significant variation with increasing amounts of carob flour (Srour et al. 2016). The results indicate that nutritional value, sensory attributes, and functional properties are improved when carob is incorporated into milk chocolate. Carob is therefore considered a suitable substitute for cocoa powder in chocolate‐based products and in the production of gluten‐free cakes formulated with banana and soy flour.

### 5.3 Pharmacological Properties of Carob

#### 5.3.1 Antimicrobial and Antifungal Properties

##### 5.3.1.1 In Vitro Studies

Extracts from *C. siliqua* have been reported to exhibit broad‐spectrum antimicrobial activity against Gram‐positive and Gram‐negative bacteria as well as several fungal strains (Hsouna et al. 2011; Brassesco et al. 2021; Darwish et al. 2021; Amrani et al. 2023). Ethanolic and methanolic extracts obtained from pods and leaves inhibited *Staphylococcus aureus*, *Bacillus subtilis*, *Escherichia coli*, and *Pseudomonas aeruginosa*, with inhibition zones ranging from 10 to 22 mm depending on extract concentration (Amrani et al. 2023). The antimicrobial activity is mainly attributed to polyphenolic compounds such as gallic acid, catechin, and quercetin derivatives, which can disrupt bacterial cell membranes and interfere with enzymatic processes (de Falco et al. 2022).

Antifungal activity has also been reported against *Candida albicans* and *Aspergillus niger*, particularly for ethanolic and acetonic extracts (Goulas and Georgiou 2020; Ghorbaninejad et al. 2023). Minimum inhibitory concentration (MIC) values generally ranged between 0.5 and 4 mg/mL. However, antimicrobial results are limited by methodological variability, as different solvents, extraction times, and microbial strains were used across studies, complicating direct comparisons. In addition, most investigations were conducted in vitro, with limited in vivo or clinical validation. Recent studies have recommended the standardization of extraction protocols and evaluation against multidrug‐resistant strains (Daoud et al. 2025).

No in vivo or clinical data are currently available for the antimicrobial and antifungal activity of *C. siliqua*; these findings should therefore be interpreted with caution as preliminary, in vitro‐only evidence.

#### 5.3.2 Antidiabetic Properties and D‐Pinitol Effects

The antidiabetic potential of *C. siliqua* has been suggested to be associated with its polyphenolic content and the presence of the cyclitol D‐pinitol (Livesey 2003; Turhan 2014; Rhimi et al. 2018; Elbouzidi et al. 2024). To avoid duplication, available evidence is summarized below according to study type.

##### 5.3.2.1 In Vitro Studies

Methanolic extracts of *C. siliqua* pods inhibited α‐amylase and α‐glucosidase activities in a concentration‐dependent manner, with half‐maximal inhibitory concentration (IC_50_) values of 0.52 and 0.83 mg/mL, respectively (Turhan 2014). D‐pinitol exhibited insulin‐mimetic activity by activating PI3K/Akt signaling pathways and enhancing glucose uptake in L6 myotubes (Azab 2022).

##### 5.3.2.2 In Vivo Animal Studies

Administration of carob pod extracts (200–400 mg/kg body weight) to streptozotocin‐induced diabetic rats was associated with reductions in fasting blood glucose levels (20%–35%) and improvements in lipid profiles after 21 days of treatment (Livesey 2003; Singh et al. 2021). Oral administration of D‐pinitol (50–100 mg/kg) increased hepatic glycogen content and enhanced antioxidant enzyme activities (Goulas and Georgiou 2020).

##### 5.3.2.3 Clinical Studies

Limited clinical trials have reported that D‐pinitol supplementation (500–600 mg/day for 4–6 weeks) reduced fasting plasma glucose and glycated hemoglobin (HbA1c) levels in patients with type 2 diabetes, with no major adverse effects observed (Azab 2022). However, these studies involved small sample sizes (N < 40) and non‐standardized extracts, limiting broader interpretation. Further randomized, double‐blind clinical trials are required to confirm these findings under controlled conditions. However, these findings should be interpreted cautiously due to limited sample sizes and lack of standardized study designs.

#### 5.3.3 Antioxidant Properties

##### 5.3.3.1 In Vitro Studies

Numerous studies have reported a potential antioxidant potential in *C. siliqua* extracts (Rizzo et al. 2004; Bahram‐Parvar and Tehrani 2011; Talibi et al. 2012; Barak and Mudgil 2014; Nawrocka et al. 2016; Srour et al. 2016). Pod and leaf extracts exhibit high total phenolic contents (20–40 mg GAE/g DW) and pronounced radical‐scavenging activity in 2,2‐diphenyl‐1‐picrylhydrazyl (DPPH) and 2,2′‐azinobis‐(3‐ethylbenzothiazoline‐6‐sulfonic acid) (ABTS) assays, comparable to standard antioxidants (Barak and Mudgil 2014). Antioxidant activity correlates closely with phenolic composition, with gallic acid, catechin, myricetin, and quercetin identified as major contributors (Nawrocka et al. 2016).

##### 5.3.3.2 In Vivo Studies

In vivo studies in animal models have shown reductions in malondialdehyde levels and increases in catalase and superoxide dismutase activities. Nevertheless, most available studies remain in vitro or preclinical and rely on crude extracts without standardization, which limits reproducibility. Future investigations should incorporate metabolite profiling and standardized extraction procedures (Dammak et al. 2024).

In addition to its antioxidant properties, carob has been explored for its potential applications in food systems. Figure 5 illustrates various uses of carob. Increasing the concentration of carob powder in white chocolate resulted in elevated levels of calcium, potassium, magnesium, and sodium, whereas iron and zinc concentrations decreased (Srour et al. 2016).

**FIGURE 5:**
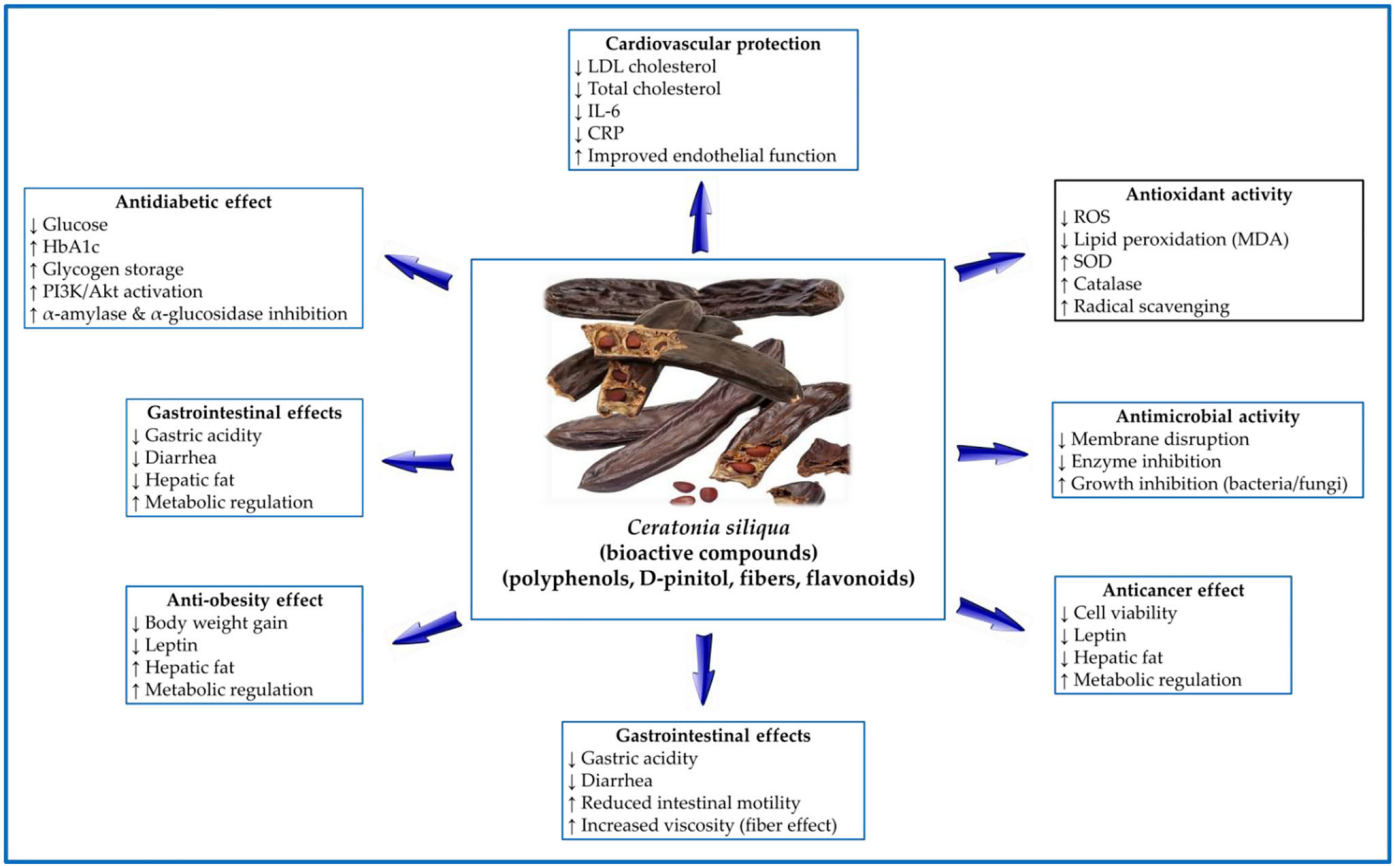
Mechanistic overview of pharmacological effects of *Ceratonia siliqua*. Akt: protein kinase B; CRP: C‐reactive protein; HbA1c: glycated hemoglobin; IL‐6: interleukin‐6; LDL: low‐density lipoprotein; MDA: malondialdehyde; PI3K: phosphoinositide 3‐kinase; ROS: reactive oxygen species; SOD: superoxide dismutase; ↑: increase; ↓: decrease.

#### 5.3.4 Antineoplastic Effects

##### 5.3.4.1 In Vitro Studies

Carob extracts have been reported to exhibit antiproliferative and pro‐apoptotic effects against several human cancer cell lines (Ennabili et al. 2000; Nayak et al. 2015; Jamous et al. 2015; O'Keefe 2016; Johansen et al. 2022). Ethanolic pod extracts reduced cell viability in melanoma (A375), colon (HT‐29), and breast (MCF‐7) cancer cell lines, with IC_50_ values ranging from 120 to 350 µg/mL (Nayak et al. 2015). Mechanistic studies have indicated apoptosis induction through mitochondrial cytochrome c release and modulation of B‐cell lymphoma 2 (Bcl‐2) family proteins (Klenow et al. 2009). These effects are largely attributed to polyphenolic constituents of *C. siliqua*, including gallic acid, catechin, epicatechin, quercetin derivatives, and proanthocyanidins, which are known to exert antiproliferative and pro‐apoptotic activities via mitochondrial and redox‐mediated pathways. However, current evidence is restricted to in vitro studies, with no animal or clinical validation of antineoplastic efficacy. Dose standardization and detailed extract characterization are required before translational application (Benyaich et al. 2025). No animal or clinical validation of antineoplastic efficacy is currently available; dose standardization and detailed extract characterization are required before any translational application can be considered (Benyaich et al. 2025).

#### 5.3.5 Anti‐Reflux Properties

##### 5.3.5.1 In Vivo Studies

Aqueous formulations of carob pod preparations have been evaluated for gastroprotective effects (Tetik and Yüksel 2014; Coviello et al. 2015; Ali et al. 2021). In animal models, high‐fiber carob powder (2‐4 g/kg) was associated with reduced gastric acidity and improved mucosal protection compared with untreated controls.

##### 5.3.5.2 Clinical Studies

Clinical studies in infants with gastroesophageal reflux reported that carob‐based thickened formulas reduced regurgitation frequency and improved feeding tolerance (Liu et al. 2023). These effects are attributed mainly to the high viscosity and water‐binding capacity of soluble fiber fractions, particularly galactomannans. Nevertheless, further studies are needed to assess long‐term safety, optimal dosage, and tolerability (Georgantelis et al. 2007).

#### 5.3.6 Antidiarrheal Effects

##### 5.3.6.1 In Vivo Studies

Methanolic and aqueous extracts of *C. siliqua* pods have been reported to alleviate diarrhea symptoms induced by castor oil or magnesium sulfate in rodent models (El‐Hilaly et al. 2003; Ouhaddou et al. 2015). The proposed mechanism involves tannins and flavonoids that inhibit intestinal motility and reduce fluid secretion (Orch et al. 2020). The effects were dose‐dependent, typically observed at doses of 100–400 mg/kg; however, the lack of standardized preparations and pharmacokinetic data limits mechanistic interpretation. No controlled clinical evaluation of antidiarrheal efficacy is currently available (Darwish et al. 2021).

#### 5.3.7 Anti‐Hyperlipidemic Effects

##### 5.3.7.1 In Vivo Studies

Carob extracts have been reported to exhibit lipid‐lowering effects in both animal and human studies (Fatima 2017; Akça et al. 2020; Dalli et al. 2021). Polyphenols and soluble fibers appear to reduce intestinal fat absorption and enhance bile acid excretion, thereby improving serum lipid profiles. In hyperlipidemic rats, ethanolic extracts administered at 200 mg/kg reduced total cholesterol by 25–30% and low‐density lipoprotein (LDL) cholesterol by approximately 20% (Fatima 2017; Akacha et al. 2026).

##### 5.3.7.2 Clinical Studies

In clinical trials, consumption of carob fiber (15 g/day for six weeks) decreased total cholesterol by 10% and LDL cholesterol by 12% without affecting high‐density lipoprotein (HDL) levels (Fatima 2017). Nevertheless, outcomes remain inconsistent due to variability in extract composition and dietary confounders (Laaraj et al. 2023). However, these effects may be influenced by dietary factors and variability in extract composition

#### 5.3.8 Anti‐Obesity Effect of Carob

##### 5.3.8.1 In Vivo Studies

Carob pulp extract was associated with anti‐obesity effects in experimental models in rats subjected to a high‐fat diet, as indicated by diminished body weight increase relative to untreated controls. This impact seems to be facilitated by various mechanisms, namely the adjustment of leptin levels and the decrease in hepatic fat accumulation. Carob treatment markedly reduced increased leptin levels caused by a high‐fat diet, indicating a partial restoration of leptin sensitivity. Despite consistent food intake, the decrease in weight gain may be ascribed to heightened energy expenditure and the hypoglycemic and hypolipidemic effects linked to enhanced leptin function. Furthermore, carob intake resulted in a reduction of liver weight, signifying the suppression of hepatic fat accumulation. The combination of carob and simvastatin further decreased leptin levels but did not improve weight loss compared to carob alone, indicating a complex interaction that necessitates additional examination (Rašković et al. 2023). It should be noted that these findings derive from a single rodent study, and the combination effect with simvastatin, a reduction in leptin without a corresponding improvement in weight loss, is not readily explained by the proposed leptin‐restoration mechanism and instead suggests that additional, uncharacterized pathways may be involved. In the absence of independent replication, standardized extract characterization, and human trials, the anti‐obesity effects of carob should currently be regarded as preliminary rather than established.

#### 5.3.9 Cardiometabolic and Cardiovascular Effects

##### 5.3.9.1 In Vivo Studies

Metabolic disorders linked to heightened cardiovascular risk necessitate specific nutritional interventions. Growing data from preclinical and clinical trials suggests that carob‐derived compounds may have cardiometabolic benefits with cardiometabolic advantages.

A murine study demonstrated that long‐term supplementation (26 weeks) with a carob‐based extract significantly mitigated metabolic changes caused by an obesogenic diet, resulting in a 17% reduction in blood glucose levels and notable enhancements in lipid profiles, including a 13% decrease in total cholesterol and a 24% reduction in LDL cholesterol (p < 0.05).

The alterations were accompanied by a notable reduction in circulating interleukin‐6 (IL‐6) levels. Carob extract treatment at the vascular level mitigated diet‐induced hypertension, reinstated endothelial function, alleviated post‐ischemic contractile dysfunction, and diminished cardiomyocyte death by over 40% (*p* < 0.05)

##### 5.3.9.2 Clinical Studies

Additionally, carob seed oil, notable for its elevated oleic acid concentration (55%–58%) and a balanced ω‐6/ω‐3 ratio of 2.3, exhibited cardiovascular advantages akin to those of olive oil. A 2023 crossover clinical trial showed that eight weeks of carob seed oil consumption led to a 21% decrease in C‐reactive protein levels in patients with cardiovascular disease (Rašković et al. 2023). These effect sizes, while promising, originate from isolated studies that have not yet been independently replicated, and the murine findings involve substantial dose extrapolation uncertainty when considered for human application. Moreover, the crossover trial on carob seed oil did not report long‐term outcomes or compare directly against olive oil in a head‐to‐head design, limiting the strength of the comparative claim. Larger, independently replicated trials with harmonized dosing and long‐term follow‐up are needed before cardiometabolic benefits can be considered clinically established.

#### 5.3.10 Critical Appraisal of Pharmacological Evidence and Knowledge Gaps

Collectively, the pharmacological literature on *C. siliqua* summarized above reveals a recurring pattern: promising bioactivity is consistently reported, but the underlying evidence base is heterogeneous and, in several respects, internally inconsistent. First, methodological variability in solvent choice, extraction time, extract standardization, and dosing makes direct comparison across studies difficult and likely explains part of the discrepancy in reported potencies (e.g., antimicrobial MIC values spanning nearly an order of magnitude, Section 5.3.1). Second, the evidentiary pyramid is inverted relative to what is needed for translational confidence: in vitro and rodent studies vastly outnumber controlled human trials, and where clinical data exist (Sections 5.3.2, 5.3.7), they are drawn from small cohorts (often *N* < 40) using non‐standardized preparations. Third, some reported effects are internally discordant, such as the dissociation between leptin modulation and weight outcomes noted in Section 5.3.8, which points to unresolved mechanistic gaps rather than a coherent mode of action. Finally, essentially no study to date has characterized the pharmacokinetics, bioavailability, or metabolic fate of carob's principal bioactive constituents (D‐pinitol, gallic acid derivatives, and proanthocyanidins) in humans, leaving a fundamental gap between the chemistry described in Section 4 and the physiological effects claimed in Section 5. Addressing these gaps will require (i) harmonized, validated extraction and characterization protocols across laboratories, (ii) adequately powered, randomized, double‐blind clinical trials, and (iii) pharmacokinetic and bioavailability studies to establish a causal, dose‐defined link between carob constituents and the health outcomes currently supported only by association.

## 6 Industrial Applications of *C. siliqua* L

Carob (*C. siliqua* L.) is a versatile industrial crop whose derivatives, particularly locust bean gum (LBG, E410), are applied across multiple sectors, including food technology, pharmaceuticals, and cosmetics. In parallel, increasing attention has been directed toward safety, regulatory compliance, and sustainability aspects of carob production and processing, in line with global trends toward eco‐efficient and health‐oriented bioproducts. This section provides an updated overview of industrial applications, regulatory considerations, and agronomic sustainability factors associated with carob.

### 6.1 Food, Pharmaceutical, and Cosmetic Uses

Carob and its derivatives have attracted industrial interest due to their multifunctional physicochemical and nutritional properties. The endosperm of *C. siliqua* seeds is the source of locust bean gum (LBG), which is widely used as a thickening, stabilizing, and gelling agent in the food industry (Son et al. 2010; Custódio et al. 2011; Krokou et al. 2019). LBG improves texture and viscosity in dairy desserts, ice creams, and bakery fillings and prevents syneresis in frozen products. It also exhibits strong synergistic effects with other hydrocolloids, such as xanthan gum and carrageenan, thereby enhancing gel strength and stability. In this context, it has been reported an applied study highlighting the technological potential of carob‐derived ingredients in food formulations, focusing on the development of carob‐enriched ice cream in which carob powder and LBG were incorporated as functional components to partially replace conventional stabilizers and lipid fractions.

In the pharmaceutical sector, carob gum is used as an excipient, binder, and controlled‐release polymer, facilitating drug delivery and improving bioavailability (Zhu et al. 2021). Its non‐toxicity, biocompatibility, and mucoadhesive properties make it suitable for ophthalmic and oral formulations. In cosmetic products, carob polysaccharides serve as natural emollients, moisturizers, and thickeners in creams, gels, and hair‐care products, providing smooth texture and improved hydration stability (Tadros 2013; Saeedabad et al. 2024).

By‐products derived from carob pods, including pulp and seed husks, are increasingly valorized as ingredients for functional foods, dietary supplements, and feed additives. These by‐products contain phenolic antioxidants, D‐pinitol, and soluble fibers that contribute to product functionality and oxidative stability (Dahmani et al. 2025).

### 6.2 Food Processing Technologies

Post‐harvest processing strongly determines the final functional quality of carob‐derived ingredients. Conventional drying and roasting remain the dominant industrial steps, but emerging non‐thermal and controlled‐thermal technologies offer potential to better preserve the phenolic and D‐pinitol content that roasting is known to diminish. Ultraviolet‐C (UVC) radiation, already established as an emerging tool for microbial disinfection of food products, represents a promising non‐thermal alternative to extend the shelf life of carob powder and pulp‐derived ingredients without the phenolic losses associated with roasting (Singh et al. 2021). Similarly, encapsulation and spray‐drying technologies, widely used to stabilize plant‐derived polyphenols in other food matrices, represent an underexplored opportunity for carob phenolics and D‐pinitol, and warrant dedicated investigation given the compositional variability.

### 6.3 Functional Ingredient Development

Carob by‐products pulp residues after gum extraction, seed husks, and germ meal are increasingly considered for functional‐ingredient development rather than treated as processing waste. Carob leaves have been characterized as a source of phenolic antioxidants with demonstrated vasorelaxant activity, supporting their potential incorporation into functional food and nutraceutical formulations beyond the pod pulp that dominates current industrial use (Dahmani et al. 2025). Realizing this potential as standardized functional ingredients (e.g., defined‐dose phenolic‐rich powders or extracts) will require extraction standardization.

### 6.4 Structure‐Function Relationships

The functional behavior of carob‐derived compounds is closely tied to their molecular structure. Locust bean gum's technological performance derives from its galactomannan backbone, in which the ratio of unsubstituted to substituted mannose regions governs its capacity to hydrate, thicken, and form synergistic gels with other hydrocolloids such as xanthan gum and carrageenan (Dionísio and Grenha 2012). Analogous structure‐function relationships are expected for carob's phenolic constituents, whose antioxidant and enzyme‐inhibitory activities are governed by hydroxylation pattern and glycosylation, and for D‐pinitol, whose cyclitol structure underlies its insulin‐mimetic signaling activity. However, unlike locust bean gum (LBG), structure‐function relationships for carob's phenolic and cyclitol constituents remain largely inferred from general phytochemical principles rather than established through carob‐specific structure‐activity studies, representing a clear research priority.

### 6.5 Food Formulation and Product Development

Beyond LBG's established role as a thickening and stabilizing agent, carob powder and pulp are increasingly incorporated as functional ingredients in product formulations such as carob‐enriched ice cream, where carob powder and LBG partially replace conventional stabilizers and lipid fractions, and carob‐enriched confectionery such as white chocolate, where carob‐derived antioxidants are used to enhance oxidative stability during storage. Successful formulation requires balancing the astringency and color contributed by carob's phenolic and tannin content against the sensory expectations of the target product category (Garau et al. 2025).

### 6.6 Sensory Properties

Carob's characteristic sweet, cocoa‐like flavor profile, attributable to its natural sugar content and a distinct volatile compound profile distinguishing it from cacao, underlies its long‐standing use as a caffeine‐free cocoa substitute (Garau et al. 2025). At the same time, higher concentrations of tannins and condensed phenolics can impart astringency and bitterness that must be managed during formulation, typically through selective use of pulp versus roasted powder fractions, or through blending strategies. Systematic sensory evaluation (descriptive analysis, consumer acceptance testing) of carob‐based products remains limited in the published literature and represents a gap relevant to the functional ingredient and product‐development applications (Soares et al. 2020).

### 6.7 Shelf‐Life and Stability Considerations

The oxidative and physical stability of carob‐derived ingredients varies by application. LBG contributes to product stability through its freeze‐thaw stability and prevention of syneresis in frozen desserts, while carob's phenolic antioxidants can extend the oxidative shelf‐life of lipid‐containing formulations, as illustrated by its use in extending storage stability in confectionery matrices. Conversely, the same phenolic fraction is itself susceptible to degradation during storage and processing, meaning packaging and storage conditions must be optimized to preserve bioactivity rather than only physical texture. The emerging biodegradable packaging applications discussed in Section 8.2 may offer a complementary route to protecting phenolic stability while addressing sustainability goals simultaneously (Ikram et al. 2023).

### 6.8 Bioaccessibility and Bioavailability of Nutrients and Phytochemicals

As already noted in the critical appraisal of pharmacological evidence, essentially no study has characterized the bioaccessibility or bioavailability of carob's principal phytochemicals (D‐pinitol, phenolic acids, and flavonoids) in humans following ingestion of real food matrices, as opposed to isolated extracts. This is a significant gap for industrial application, since the food matrix (e.g., co‐ingestion with fat in confectionery versus fiber‐rich pulp consumption) is known in general phytochemical research to substantially influence intestinal release and absorption of phenolic compounds. Encapsulation and matrix‐engineering approaches may offer a route to enhancing bioaccessibility, but this requires validation specific to carob's phenolic and cyclitol constituents rather than extrapolation from other polyphenol‐rich foods (Zannini et al. 2024).

### 6.9 Sustainability and Agronomy

The carob tree is recognized as a resilient Mediterranean crop adapted to semi‐arid and arid environments, with low irrigation requirements (300–500 mm/year) and high drought tolerance (Son et al. 2010). Under favorable agroclimatic conditions, average yields range from 2.5 to 5.0 tons of pods per hectare, and productive longevity can exceed 100 years (Martins‐Loução et al. 2024). These characteristics make *C. siliqua* a suitable candidate for sustainable agroforestry systems and for mitigating soil erosion and desertification.

By‐product valorization plays an important role in the sustainability of carob production. Seed by‐products generated during gum extraction are reused in animal feed and biomaterial formulations, while pod residues represent a valuable source of polyphenols, fibers, and natural sweeteners for functional food applications (Dahmani et al. 2025). In addition, the development of biorefinery approaches aims to utilize the entire fruit biomass, including pulp, seeds, and husks, for integrated food, feed, and bioactive compound production, thereby supporting circular economy principles (Martins‐Loução et al. 2024).

### 6.10 Industrial Uses and Processing of Locust Bean Gum (E410)

Carob seed endosperm is the source of locust bean gum (LBG), a galactomannan hydrocolloid widely employed in the food, pharmaceutical, and cosmetic industries (Krokou et al. 2019; Liu et al. 2023).

#### 6.10.1 LBG/E410 Properties

LBG is composed of a β‐(1→4)‐linked mannose backbone with α‐(1→6)‐linked galactose branches at an approximate ratio of 4:1. It exhibits high viscosity (>3000 mPa·s in a 1% solution) and strong synergistic interactions with other hydrocolloids, such as xanthan gum and carrageenan (Guo et al. 2021). Optimal hydration occurs at temperatures between 70°C and 85°C, and LBG remains stable across a potential of hydrogen (pH) range of 4–10.

#### 6.10.2 Food Applications

LBG (E410) is authorized as a thickener, stabilizer, and gelling agent in a wide range of food products, including dairy products, desserts, sauces, and beverages. It is approved by the European Food Safety Authority ([EFSA]; Regulation [EU] No 231/2012) and the U.S. Food and Drug Administration (21 CFR 172.695). Its high viscosity and freeze–thaw stability contribute to improved texture and mouthfeel in food formulations (Zhu et al. 2021).

#### 6.10.3 Pharmaceutical and Cosmetic Applications

In pharmaceutical formulations, LBG is used as a tablet binder, disintegrant, and controlled‐release matrix polymer, enhancing bioadhesion and swelling properties (Dionísio and Grenha 2012). In cosmetic products, it functions as a natural emollient and thickener in creams and gels due to its non‐toxic, biodegradable, and biocompatible nature (Fisher and Phillips 2008; Dwivedi et al. 2022; Imeson 1992).

### 6.11 Comparative Perspective With Other Mediterranean Functional Crops

*C. siliqua* is one of several Mediterranean tree crops historically valued for both nutritional and functional properties, alongside olive, almond, fig, and date palm. Traditionally, olive, carob, almond, and fig were intercropped within the same Mediterranean agroforestry systems, exploiting their complementary fruiting and resource‐use patterns; however, olive and almond cultivation was subsequently intensified through irrigation, while carob and fig were more often left within lower‐input, drought‐tolerant systems (Martins‐Loução et al. 2024).

This agronomic divergence reflects a broader functional distinction: olive and almond are primarily valued for their oil and lipid‐rich kernels, whereas carob is essentially fat‐free and instead offers a rare combination of high dietary fiber, natural sweetness, and a co‐product with no direct equivalent among the other crops discussed here.

From a phytochemical standpoint, carob's polyphenol profile is comparable in diversity to other Mediterranean functional foods rich in phenolics, such as olive (oleuropein and hydroxytyrosol) and pomegranate (ellagitannins), but is distinguished by its combined abundance of polyphenols, condensed tannins, and dietary fiber within a single low‐fat matrix (Benyaich et al. 2025). This combination positions carob less as a direct substitute for oil‐based Mediterranean functional foods (olive) and more as a complementary source of fiber‐ and polyphenol‐based functionality, alongside its established, industrially unique role as a hydrocolloid (LBG) source.

Agronomically, carob's drought tolerance and productive longevity exceeding a century are broadly comparable to and, in terms of irrigation requirements, generally lower than those of olive and almond, both of which, despite their own resilience, have historically required greater irrigation investment to sustain commercial yields (Martins‐Loução et al. 2024). This makes carob a particularly strong candidate for low‐input, marginal‐land agroforestry relative to the other crops discussed, complementing rather than displacing their established roles in the Mediterranean functional‐food landscape.

## 7 Safety and Regulations

### 7.1 Toxicology and Allergenicity

Carob and its derivatives, particularly LBG (E410), exhibit a high margin of safety based on toxicological evaluations. Animal studies have shown no evidence of mutagenicity, carcinogenicity, or teratogenicity at doses exceeding 2000 mg/kg body weight (Krokou et al. 2019). LBG is generally considered non‐allergenic and non‐sensitizing in both humans and animals, although isolated cases of mild intolerance to high‐fiber carob products have been reported, particularly in infants and individuals with irritable bowel syndrome (Azab 2022).

### 7.2 Intake Considerations and Adverse Effects

The average daily intake of LBG from food sources is estimated to range from 5 to 200 mg/kg body weight, which is well below levels associated with gastrointestinal discomfort. Excessive consumption may result in transient bloating or laxative effects due to the high soluble fiber content (Gill et al. 2021). No acute or chronic toxicity has been reported in humans.

### 7.3 Regulatory Status

LBG is listed as a permitted food additive under the European code E410 and is approved by the EFSA, the U.S. Food and Drug Administration (FDA), and the Joint FAO/WHO Expert Committee on Food Additives (JECFA). EFSA has confirmed that LBG is safe for all age groups when used in accordance with Good Manufacturing Practice (GMP). The FDA classifies LBG as Generally Recognized as Safe (GRAS) under 21 CFR 172.695, and the Codex Alimentarius includes it as a stabilizer and thickener in numerous food categories. Maximum permitted use levels generally range from 0.3% to 1%, depending on product type and national regulations (EFSA 2023). In 2022, EFSA re‐evaluated locust bean gum (E410) for use in foods for infants below 16 weeks of age and initiated follow‐up assessments for other population groups. The EFSA Panel on Food Additives and Nutrient Sources Added to Food identified a reference point of 1,400 mg/kg body weight per day, based on reduced blood zinc levels observed in a piglet study, and concluded that margins of exposure above one do not raise safety concerns under the evaluated exposure scenarios ([CSL STYLE ERROR: reference with no printed form.]).

LBG is included in the Codex Alimentarius as a permitted food additive and is assigned the European code E410. Its use is authorized in more than 70 countries worldwide. Industrial specifications typically require a galactomannan content of at least 75%, moisture levels below 12%, and a pH range of 5.4–7.2 for a 1% aqueous solution (Dunford et al. 2023).

Overall, *C. siliqua* exhibits a broad range of biological and industrial properties. While most bioactivity studies remain in vitro or preclinical and require further standardization and clinical validation, its industrial relevance, particularly through LBG (E410), is supported by well‐defined physicochemical parameters, established safety profiles, and broad regulatory acceptance.

## 8 Future Perspectives

### 8.1 Emerging Analytical and Metabolomic Approaches

Future characterization of *C. siliqua* should move beyond the targeted, single‐class analyses (HPLC/UPLC‐UV or MS focused on a handful of known phenolics) that dominate the current literature (Section 4) toward untargeted, multi‐platform metabolomics (LC‐MS/MS and GC‐MS combined with multivariate statistics) capable of resolving the full metabolite complement across pods, seeds, and leaves and linking specific metabolite signatures to genotype, geographic origin, and harvest conditions. Coupling metabolomics with transcriptomic and proteomic data would help clarify the biosynthetic pathways underlying the variability noted throughout Section 5 and support the development of chemical fingerprints for cultivar authentication and quality control, an area with essentially no published data to date. In parallel, emerging non‐destructive analytical tools, including near‐infrared (NIR) spectroscopy and machine‐learning‐assisted chemometric classification, could enable rapid, field‐deployable screening of carob pod quality and bioactive content, reducing reliance on slow, solvent‐intensive extraction‐based assays.

### 8.2 Valorization Strategies and Circular Bioeconomy Applications

Beyond the established use of locust bean gum (Section 6), substantial valorization potential remains in carob's secondary fractions. Pod pulp residues after gum extraction, seed husks, and germ meal are currently under‐exploited but represent candidate feedstocks for cascading biorefinery schemes that sequentially recover polyphenols, dietary fiber, and fermentable sugars before directing the remaining lignocellulosic residue toward biochar, animal feed, or bioenergy production. Carob‐derived galactomannans and polyphenol‐rich fractions also show promise as components of biodegradable packaging films and edible coatings, offering a renewable alternative to petroleum‐based plastics and aligning with zero‐waste, circular bioeconomy models increasingly demanded by the food and cosmetics industries. Realizing this potential will require techno‐economic and life‐cycle assessments to determine whether such valorization pathways are viable at an industrial scale relative to conventional processing routes.

### 8.3 Research Priorities

Based on the gaps identified throughout this review, we propose the following priorities for future research: (i) development and adoption of harmonized, validated extraction and analytical protocols, together with certified reference materials, to enable cross‐study comparability; (ii) adequately powered, randomized, double‐blind clinical trials with standardized carob extracts to substantiate the antidiabetic, antihyperlipidemic, and cardiometabolic effects currently supported mainly by preclinical data; (iii) pharmacokinetic and bioavailability studies of key constituents (D‐pinitol, gallic acid derivatives, and proanthocyanidins) in humans; (iv) integration of multi‐omics approaches to establish structure–activity relationships and cultivar‐specific chemical fingerprints; and (v) techno‐economic evaluation of biorefinery and circular‐economy valorization pathways to guide industrial investment decisions.

## 9 Conclusions

*C. siliqua* L. emerges as a multifunctional Mediterranean species with demonstrated nutritional, pharmacological, and industrial relevance, underpinned by a rich matrix of polyphenols, dietary fiber, D‐pinitol, and galactomannans. Its resilience to arid conditions further positions it as a strategic crop for sustainable agriculture. However, as detailed in Sections 5.3.10 and 8, translating this potential into validated applications will depend on resolving methodological heterogeneity, expanding clinical validation, and pursuing the analytical and valorization priorities outlined above. With continued interdisciplinary research spanning phytochemistry, clinical pharmacology, and process engineering, carob is well positioned to bridge traditional ethnobotanical use and modern sustainable bioindustry.

## Author Contributions

**Anis Ben Hsouna**: conceptualization, writing – original draft, methodology. **Ravendra Kumar**: conceptualization, writing – original draft, methodology. **Manjul Gondwal**: conceptualization, writing – original draft, methodology. **Shivangi Chamoli**: methodology, conceptualization, writing – original draft. **Bhanu Pratap Singh Gautam**: conceptualization, methodology, writing – original draft. **Simranpreet Kaur**: conceptualization, methodology, writing – original draft. **Rakesh Kumar Verma**: conceptualization, methodology, writing – original draft. **Piyush Kumar**: conceptualization, methodology, writing – original draft. **Monika Michalak**: conceptualization, methodology, writing – original draft. **Boutheina Ben Akacha**: conceptualization, methodology, writing – original draft. **Wirginia Kukula‐Koch**: conceptualization, methodology, writing – original draft. **Wojciech Koch**: conceptualization, methodology, writing – original draft. **Faical Brini**: conceptualization, methodology, writing – original draft. **Wissem Mnif**: conceptualization, methodology, writing – original draft. **Rania Ben Saad**: conceptualization, methodology, writing – original draft. **Stefania Garzoli**: conceptualization, writing – original draft, methodology. **Miroslava Kačániová**: conceptualization, methodology, writing – original draft.

## Funding

This research was supported by the Deanship of Graduate Studies and Scientific Research at the University of Bisha‐Saudi Arabia, through the Fast‐Track Research Support Program. This study was supported by a grant from the Tunisian Ministry of Higher Education and Scientific Research (Program Contract 2022–2026).

## Conflicts of Interest

The authors declare no conflicts of interest.

## Data Availability

The data are available upon request from the authors.
